# Isolation and propagation of an Egyptian *Theileria annulata* infected cell line and evaluation of its use as a vaccine to protect cattle against field challenge

**DOI:** 10.1038/s41598-024-57325-2

**Published:** 2024-04-12

**Authors:** Amira AL-Hosary, Ahmed M. Radwan, Laila S. Ahmed, Sary Kh. Abdelghaffar, Susanne Fischer, Ard M. Nijhof, Peter-Henning Clausen, Jabbar S. Ahmed

**Affiliations:** 1 Department of Animal Medicine (Infectious Diseases), Faculty of Veterinary Medicine, Assiut University, Assiut, 71526 Egypt; 2 Field Veterinarian, EL-Minia’s Veterinary Directorate, EL-Minia, Egypt; 3 Department of Pathology and Clinical Pathology, Faculty of Veterinary Medicine, Assiut University, Assiut, 71526 Egypt; 4 Department of Pathology and Clinical Pathology, School of Veterinary Medicine, Badr University in Assiut, Assiut, Egypt; 5 Institute of Infectology, Friedrich-Loeffler-Institut, Südufer 10, Insel Riems, 17943 Greifswald, Germany; 6 Institute of Parasitology and Tropical Veterinary Medicine, Freie Universität Berlin, 14163 Berlin, Germany; 7 Veterinary Center for Resistance Research, Freie Universität Berlin, 14163 Berlin, Germany

**Keywords:** *Theileria annulata*, Cell line, Cattle, Vaccine, Egypt, Biological techniques, Immunology

## Abstract

Tropical theileriosis is an important protozoan tick-borne disease in cattle. Vaccination using attenuated schizont-infected cell lines is one of the methods used for controlling the disease. This study describes the production of attenuated schizont-infected cell lines from Egypt and an evaluation of its use as a vaccine to protect calves against clinical disease upon field challenge. Two groups of exotic and crossbred male calves were divided into vaccinated and control groups. The vaccinated groups were inoculated with 4 ml (1 × 10^6^ cells/ml) of the attenuated cell line. Three weeks after vaccination, calves of both groups were transported to the New Valley Governorate (Egyptian oasis) where they were kept under field conditions and exposed to the natural *Theileria annulata* challenge. All animals in the control group showed severe clinical signs and died despite treatment with buparvaquone, which was administered after two days of persistent fever due to a severe drop in packed cell volume (PCV). Animals in the vaccinated group became seropositive without developing severe clinical signs other than transient fever. Post-mortem examinations revealed enlarged and fragile lymph nodes, spleen, and liver with necrosis and hemorrhages. These findings indicate that the Egyptian attenuated cell line was successful in protecting both exotic and crossbred animals against tropical theileriosis under field conditions.

## Introduction

Bovine theileriosis caused by *Theileria annulata* (*T. annulata*) is an important disease of cattle in subtropical and tropical regions where vector ticks of the genus Hyalomma occur^1–3^. The infection is transmitted from ticks to their bovine host through tick saliva containing *T. annulata* sporozoites. The sporozoites enter the host's bloodstream and travel to the lymph nodes. Here they infect and transform the lymphocytes through asexual reproduction into the schizont stage. The schizonts divide rapidly, producing many merozoites. The schizont can regulate the cell function and programmed cell death through the secretion of proteins directly into the cell cytoplasm, affecting cell signaling and function. This results in cell transformation, making the host cell immortal so that it proliferates continuously. The merozoites then infect other lymphocytes and continue the cycle of asexual reproduction. The merozoites are released into the bloodstream and infect erythrocytes (red blood corpuscles, RBCs). Inside the RBCs, the merozoites continue to develop and divide, producing more merozoites. This stage is responsible for the clinical signs of tropical theileriosis. Some of the merozoites differentiate into male and female gametocytes and when the tick feeds on an infected animal, it ingests the gametocytes, which then undergo sexual reproduction in the tick's gut. The fertilized eggs develop into sporozoites, which migrate to the tick's salivary glands, ready to infect the next host^4,5^.

The distribution of the parasite into daughter cells is accompanied by a tight association with the mitotic apparatus of the host cell^6^. *T. annulata* transforms leucocytes, loses virulence after long-term passage and is used as a live vaccine in endemic countries^7,8^. In Egypt, clinical cases of theileriosis have been reported in different breeds of cattle and buffaloes (*Bubalus bubalis*) all over the Nile valley and other localities such as the Delta region of the Nile valley, Upper Egypt and the Egyptian oasis^9,10^. Early recognition of clinical cases plays an important role in the treatment and control of the disease^11,12^. During the acute phase when relatively high parasitemia is found, it can be easily diagnosed by using Giemsa-stained blood and lymph node aspiration smears^2,13,14^. In the late chronic or carrier phase, parasitemia decreases to microscopically undetectable levels and diagnosis depends mainly on serological and molecular tests^15,16^. In Egypt, the lack of reliable integrated control programmes that include accurate diagnosis, tick control, chemotherapy and vaccination, which has hampered control of the disease^17^. On the other hand, successful vaccination using live attenuated schizont infected cell lines has been reported in many countries in North Africa, the Middle East and South Asia^11,12,18,19^. However, concerns that parasite isolates may not be identical in antigenic composition or virulence have led to the propagation of local *T. annulata*-infected cell lines and evaluation of their efficacy under experimental and field challenges in each country or region. This was the impetus for the current study, which aimed to produce a local vaccine for Egypt by isolating and attenuating infected peripheral blood leukocytes (PBMC) from naturally infected cattle, followed by an evaluation of its potential as a vaccine to protect cattle against *T. annulata* exposure in the field.

## Results

### Cell lines

*T. annulata* schizont-infected cell line was prepared from leukocytes isolated from both heparinized blood and lymph node aspirates. The DNA extracts from the cultures were tested using polymerase chain reaction (PCR) targeting the small subunit (SSU) 18S rRNA (18S rRNA) gene and Reverse Line Blot (RLB) assays, followed by sequencing. The results were positive for *T. annulata* but not for other tick-borne pathogens. The partial 18S rRNA gene sequence of the isolate was submitted to GenBank and can be retrieved using accession number MN704769.

The appropriate dose and passage for the vaccination trial at a dose of 4 ml (1 × 10^6^ cells/ml) was found to be the most immunogenic dose, inducing the strongest immune response with no adverse effects or clinical signs. It protected the vaccinated animals during the natural challenge under field conditions, which developed no clinical signs other than transient fever until slaughter (Table 4).

### Vaccination trial

**Figure 1:**
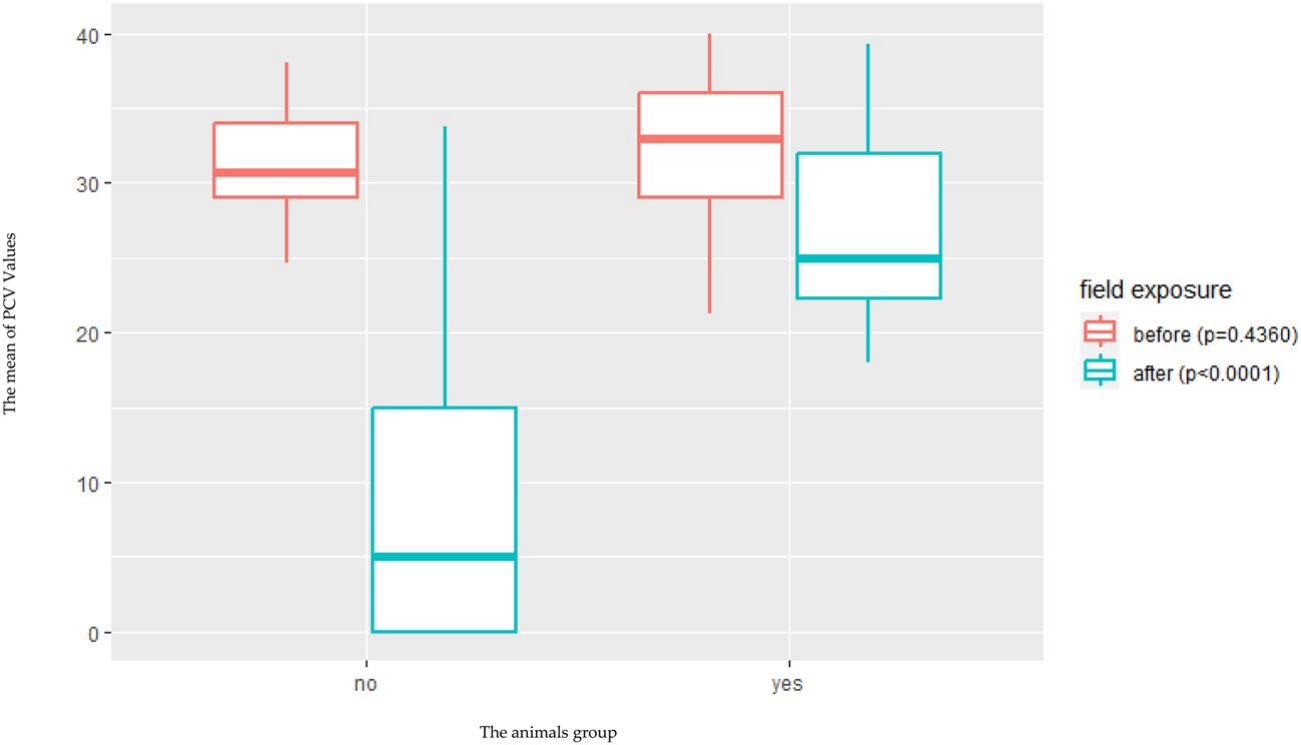
Statistical analysis for the PCV results in both groups before and after challenge. X-axis represents and Y-axis represents the animals’ group (No = represents the mean distribution of PCV for not vaccinated calves and yes = represents the mean distribution of PCV for vaccinated calves). P-value before challenge (p = 0.4360) and P-value after challenge (p < 0.0001).

**Figure 2:**
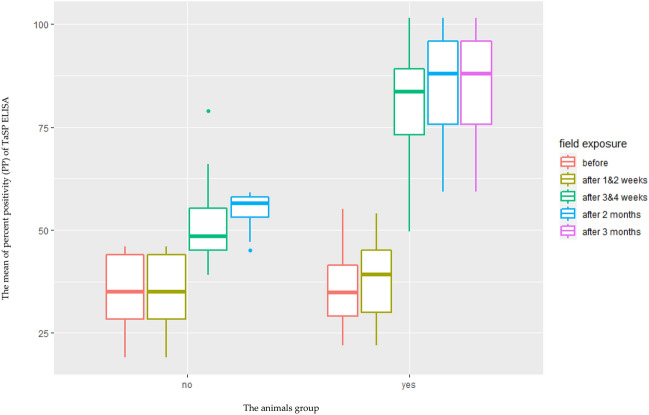
Statistical analysis for the TaSp ELISA results in both groups before and after challenge. X-axis represents the mean of percent positivity (PP) of TaSP ELISA, and Y-axis represents the animals’ group (no = represents the mean distribution of Abs’ PP in not vaccinated calves before and after the challenge and yes = represents the mean distribution of Abs’ PP in vaccinated calves before and after the challenge) (P < 0.0001).

**Figure. 3:**
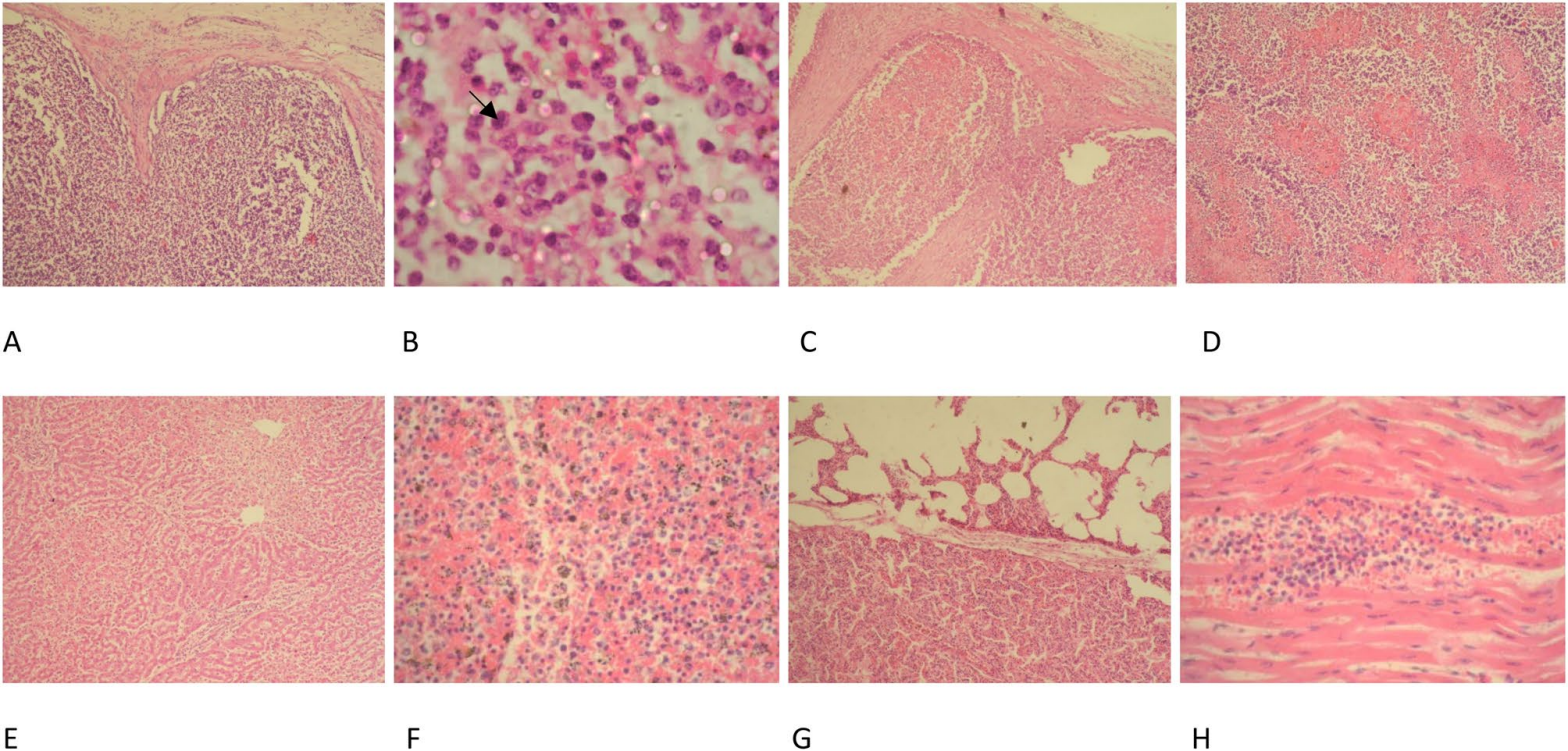
Microscopic finding of the lesions founded in the dead control animals, (**A**) Lymph node section from cattle with chronic theileriosis showing only depletion of lymphoid elements in the cortex. H&E. X10. (**B**) Lymph node section from cattle with acute theileriosis showing extensive necrosis and hemorrhage in the medulla with presence of schizont-infected cells (arrows). H&E. X100. (**C**) Lymph node section from cattle with acute theileriosis showing extensive necrosis and hemorrhage in the cortex. H&E. X10. (**D**) Lymph node section from cattle with acute theileriosis showing extensive necrosis and hemorrhage in the medulla. H&E.X10 (**E**) Liver section from cattle with acute theileriosis showing multiple necrotic foci. H&E.X10 (**F**) Spleen section from cattle with acute theileriosis showing necrotic foci associated with hemorrhage and hemosidrosis. H&E. X40. (**G**) Lung section from cattle with chronic theileriosis showing alveolar emphysema and lobular interstitial pneumonia. H&E. X10. (H) Myocardial section from cattle with acute theileriosis showing necrotic foci associated with hemorrhage and lymphoid cell reaction. H&E. X40.

All animals were tested negative for *T. annulata* infection prior to vaccination by using polymerase chain reaction (PCR) targeting the small subunit (SSU) 18S rRNA gene (18S rRNA) and *T. annulata* major merozoite piroplasm surface antigen gene (Tams1), and by *T. annulata* surface protein ELISA (TaSp ELISA). After vaccination and before field challenge, only animals of the vaccinated group became PCR-positive two weeks after vaccination, and TaSp antibodies were detected 4–5 weeks post vaccination. None of the animals developed clinical disease. Piroplasm or schizont stages were not observed microscopically in any of the vaccinated animals. Within a few hours of the start of the field challenge, all animals were infested with ticks, mainly *Hyalomma* spp, especially *Hyalomma excavatum*, in addition to some *Rhipicephalus annulatus* ticks which detected later during the challenge period. The ticks mainly attached to the scrotum, dewlap, inner side of the thighs and tail. The first clinical signs indicative for *T. annulata* infections were observed after 5 to 12 days post tick infestation. These signs included fever with body temperatures of up to 41.5 °C, ocular discharge, enlargement of superficial lymph nodes, of the prescapular and prefemoral lymph nodes, various degrees of respiratory signs including nasal discharge and coughing, pale mucous membranes, jaundice, and diarrhea. The clinical signs were more severe in the control group compared to the vaccinated group. Animals in the control group became anorexic and died despite treatment with buparvaquone at the recommended dose of 2.5 mg/kg body weight. The treatment was applied after animals had persistent fever for two days. The PCV ranged from 26.5 to 40% before challenge in both vaccinated and control groups and dropped after field challenge to 18–30% in vaccinated animals and 3–34% in the control group (Fig. 1, Tables 1, 2). The TaSp antibodies increased in all animals. The antibodies’ titers before and after challenge being statistically different in the vaccinated group when compared to the control group (Fig. 2). Animals of control groups became positive by both Tams-1 and 18S rRNA PCR within ten days post challenge, and this was confirmed by sequencing. The obtained sequences were identical, and we submitted one of them as an example to the GenBank and it is available under accession number MN704771. Post-mortem examinations revealed that the body cavities of all diseased animals were filled with yellow straw-colored fluid and that petechial hemorrhages were widely distributed on the serous membranes. The lymph nodes, liver and gall bladder were enlarged, edematous and fragile with petechial hemorrhages. Extensive necrosis and hemorrhages were seen in the medulla of the affected lymph nodes. Schizont-infected cells that represent the schizont stages of *T. annulata* were confirmed microscopically in the lymph nodes of animals with acute theileriosis. Multiple necrotic foci, edema and foci of lymphoid cell reaction were reported in the liver sections. The spleens were enlarged, edematous and fragile with petechial hemorrhages. Multiple necrotic foci and depletion of lymphoid elements in both white and red pulps were noticed, associated with hemorrhage and hemosiderosis. Both lungs were emphysematous and dark in color with several areas of hepatization. Also, frothy exudate was found in the bronchi and bronchioles. Microscopic examination revealed alveolar emphysema, lobular interstitial pneumonia, and foci of lymphoid cell reaction. Myocardial section showed multiple necrotic foci associated with hemorrhage and interstitial lymphoid cell reaction (Fig. 3).

**Table 1:** Follow up Parameters before and after challenge in Exotic breed calves including start of fever (day post infection), maximum temperature (of fever), percentage of PCV before challenge and lowest after challenge. Additionally, the maximum parasitemia, treated or not and fate are displayed.

Animal ID	Group	Breed	Start of fever	Maximum temperature	PCV/Hematocrit before challenge	Lowest PCV observed/ Hematocrit	Maximum parasitemia	Treatment	Fate
6947	Vaccinated	Exotic	Day 10	39.9 °C (day 10)	27.9%	19%	Post. (0.0004)	No Treatment	Survived
6881	Vaccinated	Exotic	Day 9	40 °C (day 10)	28.2%	20%	Post. (0.0008)	No Treatment	Survived
6909	Vaccinated	Exotic	Day 8	40.1 °C (day 10)	32.4%	20%	Post. (0.00064)	No Treatment	Survived
1 V	Vaccinated	Exotic	Day 7	39.8 °C (day 7)	32.8%	22%	Post. (0.0004)	No Treatment	Survived
2 V	Vaccinated	Exotic	Day 10	39.9 °C (day 10)	33.6%	22%	Pos. (0.0004)	No Treatment	Survived
3 V	Vaccinated	Exotic	Day 10	39.9 °C (day 10)	28.1%	21%	Post. (0.0008)	No Treatment	Survived
4 V	Vaccinated	Exotic	Day 10	40.5 °C (day 12)	32%	21%	Pos. (0.00056)	No Treatment	Survived
5 V	Vaccinated	Exotic	Day 11	40 °C (day 11)	33. 8%	20%	Post. (0.0004)	No Treatment	Survived
6 V	Vaccinated	Exotic	Day 10	39.6 °C (day 10)	26.5%	21%	Post. (0.00064)	No Treatment	Survived
7 V	Vaccinated	Exotic	Day 10	40 °C (day 10)	33%	18%	Post. (0.0004)	No Treatment	Survived
8 V	Vaccinated	Exotic	Day 9	40.2 °C (day 10)	37%	23%	Post. (0.0008)	No Treatment	Survived
9 V	Vaccinated	Exotic	Day 9	40.7C (day 10)	40%	19%	Post. (0.0008)	No Treatment	Survived
6907C	Control	Exotic	Day 7	41.5 °C (day 8)	28.5%	5%	Pos. (0.0016)	Treated	Died at day 28
6911C	Control	Exotic	Day 7	41 °C (day 8)	28.5%	7%	Pos. (0.0012)	Treated	Died at day 21
6913C	Control	Exotic	Day 10	41.5 °C (day 10)	28.2%	3%	Pos. (0.00128)	Treated	Died at day 56
1C	Control	Exotic	Day 8	41 °C (day 8)	36%	5%	Pos. (0.0016)	Treated	Died day 70
2C	Control	Exotic	Day 7	41 °C (day 8)	34%	7%	Pos. (0.0016)	Treated	Died at day 35
3C	Control	Exotic	Day 7	40.5 °C (day 8)	33%	8%	Pos. (0.00144)	Treated	Died at day 28
4C	Control	Exotic	Day 7	41 °C (day 7)	35%	5%	Pos. (0.00152)	Treated	Died at day 28
5C	Control	Exotic	Day 7	41 °C (day 7)	33.5%	7%	Pos. (0.0016)	Treated	Died at day 21
6C	Control	Exotic	Day 7	41 °C (day 10)	36.2%	13%	Pos. (0.002)	Treated	Died at day 28
7C	Control	Exotic	Day 7	40 °C (day 10)	29.5%	5%	Pos. (0.037)	Treated	Died at day 28
8C	Control	Exotic	Day 5	40.6 °C (day 8)	33%	7%	Pos. (0.002)	Treated	Died at day 21
9C	Control	Exotic	Day 8	40 °C (day 8)	28.2%	13%	Pos. (0.002)	Treated	Died at day 28

**Table 2:** Follow up Parameters before and after challenge in Crossbreed calves including start of fever (day post infection), maximum temperature (of fever), percentage of PCV before challenge and lowest after challenge. Additionally, the maximum parasitemia, treated or not and fate are displayed.

Animal ID	Group	Breed	Start of fever (day after tick infestation)	Maximum temperature (°C)	PCV/Hematocrit before Challenge	Lowest PCV observed/Hematocrit	Maximum parasitemia	Treatment	Fate
256,255	Vaccinated	Crossbreed	Day 6	39.9 °C (Day 7)	40%	20,5%	Post. (0.0004)	No treatment	Survived
256,310	Vaccinated	Crossbreed	Day 6	40 °C (Day 10)	34.2%	19%	Post. (0.0004)	No treatment	Survived
239,967	Vaccinated	Crossbreed	Day 5	40.1 °C (Day 11)	31.1%	20%	Post. (0.00048)	No treatment	Survived
251,243	Vaccinated	Crossbreed	Day 8	39.8 °C (Day 7)	30.1%	20%	Post. (0.0004)	No treatment	Survived
10 V	Vaccinated	Crossbreed	Day 11	39.9 °C (Day 7)	33%	20%	Post. (0.0004)	No treatment	Survived
11 V	Vaccinated	Crossbreed	Day 10	39.9 °C (Day 7)	34%	24%	Post. (0.0008)	No treatment	Survived
12 V	Vaccinated	Crossbreed	Day 8	40.5 °C (Day 9)	33%	25%	Post. (0.0008)	No treatment	Survived
13 V	Vaccinated	Crossbreed	Day 7	40 °C (Day 8)	37%	29%	Post. (0.0008)	No treatment	Survived
14 V	Vaccinated	Crossbreed	Day 8	39.6 °C (Day 10)	39%	21%	Post. (0.00064)	No treatment	Survived
15 V	Vaccinated	Crossbreed	Day 9	40 °C (Day 8)	36%	30%	Post. (0.0008)	No treatment	Survived
16 V	Vaccinated	Crossbreed	Day 10	40.2 °C (Day 13)	37%	21%	Post (0.00056)	No treatment	Survived
17 V	Vaccinated	Crossbreed	Day 10	40.7(Day 12)	35%	30%	Post. (0.0008)	No treatment	Survived
256056C	Control	Crossbreed	Day 5	41 °C (Day 6)	34.4%	3%	Pos. (0.003)	Treated	Dead at 70 days
256271C	Control	Crossbreed	Day 5	40.9 °C (Day 7)	33%	4%	Pos. (0.002)	Treated	Dead at 70 days
259577C	Control	Crossbreed	Day 7	40.9 °C (Days 7)	29.7%	5%	Pos. (0.002)	Treated	Dead at 77 days
253027C	Control	Crossbreed	Day 6	41 °C (Day 6)	32.4%	4%	Pos. (0.002)	Treated	Dead at 63 days
202133C	Control	Crossbreed	Day 6	41 °C (Day 6)	27.3%	5%	Pos. (0.0033)	Treated	Dead at 63 days
258165C	Control	Crossbreed	Day 10	40.5 °C (Day 12)	31.3%	5%	Pos. (0.0033)	Treated	Dead at 70 days
10C	Control	Crossbreed	Day 10	41.5 °C (Day 14)	34.4%	5%	Pos. (0.002)	Treated	Dead at 49 days
11C	Control	Crossbreed	Day 10	41 °C (Days 10)	33%	4%	Pos. (0.002)	Treated	Dead at 49 days
12C	Control	Crossbreed	Day 10	41.5 °C (Day 10)	29.7%	9%	Pos. (0.002)	Treated	Dead at 42 days
13C	Control	Crossbreed	Day 10	40 °C (Day 10)	32%	7%	Pos. (0.002)	Treated	Dead at 42 days
14C	Control	Crossbreed	Day 10	40.6 °C (Day 16)	37.3%	9%	Pos. (0.002)	Treated	Dead at 42 days
15C	Control	Crossbreed	Day 10	40 °C (Day 10)	36%	9%	Pos. (0.002)	Treated	Dead at 42 days

### Co-detection of other TBPs after challenge

RLB assays were performed on blood samples collected from animals before and after vaccination and after challenge. The results revealed that all animals were negative for tick-borne pathogens at the start of the study. Only vaccinated animals turned positive for *T. annulata* after inoculation of the attenuated cell line. After field challenge all animals became positive for *T. annulata*, some of them also tested positive for other tick-borne pathogens (Table 3). All co-infections were considered as subclinical because the blood smears and 18S rRNA PCR were negative for all pathogens except for *T. annulata* which confirmed with Tams-1 PCR and sequencing.as All the obtained sequences were identical, we submitted only one of them as an example to the GenBank and it is available under accession number MN704770.

**Table 3:** Co-infection with other Tick-Borne Pathogens in both vaccinated and control animals of both breeds:

Pathogens	Vaccinated Animals	Control Animals
*T. annulata*	24	24
*Babesia bovis*	1	3
*T. annulata* + *Anaplasma marginale*	10	16

## Discussion

Tropical theileriosis is a major obstacle to the development of livestock production in most tropical and subtropical countries, including Egypt. Control of ticks and tick-borne diseases in Egypt depends largely on the use of chemical acaricides. However, the development of resistance to acaricides is limiting this approach. This situation creates the need for other effective control measures^20,21^. Vaccination against tropical theileriosis using an attenuated cell culture vaccine is an interesting opportunity for the control and/or eradication of tropical theileriosis. This approach has been previously reported in several other North African countries, including Sudan and Tunisia, but not in Egypt^11,18,22–24^. In this study, we described the development of an attenuated cell line of *T. annulata* (Egyptian strain). This Egyptian strain was prepared and attenuated according to previously published protocols^23,24^. The quality of this attenuated cell line and the appropriate dose were also determined based on previous trials and studies which concluded that the infected cell line at passage 85 was sufficient to be used as a vaccine dose. This passage was close to the attenuation passage in the Sudanese cell line^24^ and some Tunisian cell lines^18,22^. On the other hand, it was far from other Tunisian cell lines, which need more than two hundred passages to be attenuated and ready to use, and this finding confirmed that each locality should have its own local strain, as described before in many studies^11,18,22–24^. After vaccination and field challenge, both control and vaccinated animals were infested with ticks. All collected ticks were identified as *Hyalomma excavatum*, which is considered one of the main biological vectors of *T. annulata* in Egypt and *Rhipicephalus annulatus*, which may accidentally carry and transmit it mechanically by feeding on infected or carrier animals. This finding was in agreement with previous finding in Egypt which concluded that both previously mentioned species are the most common species in Egypt and especially in the Egyptian oasis^3^. The recorded clinical signs were more severe in the control group compared to the vaccinated group. Control animals gradually became anorexic and died. This finding was in agreement with some previous studies that specified the same clinical signs for *T. annulata* vaccination and challenge trials^1,9,11,25^. PCV results in both vaccinated and control groups did not significantly differ before challenge (p = 0.4360) using Generalized linear model (GLM) but in contrast, two to three weeks after the challenge the PCV dropped significantly in the control groups when compared with the vaccinated group (p < 0.0001). This result indicates a heavy infection with *T. annulata* in the control group (Fig. 1). This finding is supported by previous finding which indicated severe drop in the PCV in case of *T. annulata* infection and its co-infection due to the increase in erythrophagocytosis, lysis of parasitized erythrocytes due to multiplication of the parasite and subsequent removal by the reticuloendothelial system, and the increase in osmotic fragility of parasitized erythrocytes^26–28^. The antibodies’ titers raised in all vaccinated animals according to the results obtained by using TaSP-ELISA assay when compared with the data collected from control group. This finding indicated that the attenuated cell line can enhance the production of statistically significant titer of the protective antibodies without significant clinical infection (P > 0.0001)^22,24^. The protection rate in the vaccinated groups was 100% and it is closely similar to the finding recorded in China which ranged from 99.5 to 99.9%^29–35^. Co-infection with other tick-borne pathogens was detected through the RLB assay which is more sensitive when compared to blood smears, PCR, and nested PCR, suggesting that all co-infection occurred at low levels^2,36^. The higher co-infections in the unvaccinated (control) group compared to the vaccinated group could be attributed to the heterologous or non-specific immunological effects (NSE) that can be induced when using live attenuated vaccines. These NSE have been described in several studies showing that innate immune cells, such as natural killer cells, monocytes, and macrophages, can provide non-specific protection against certain infections in vaccinated animals receiving live attenuated vaccines. This process referred to as "trained immunity", is regulated by epigenetic reprogramming of innate immune cells and is responsible for the protective, non-specific effects induced by vaccines^37–40^. As all co-infections reported in the control groups were due to intracellular pathogens such as Babesia and Anaplasma, we suggested that this NSE could play a critical role in preventing these co-infections in vaccinated animals by recognizing infected RBCs by various receptors of the host innate immune system and activating cytokines and phagocytosis, which play a crucial role in clearing parasitized RBCs as previously mentioned in several studies^41,42^. The post-mortem lesions observed in died control animals were similar to the previously mentioned lesions in studies that investigated fatal cases of *T. annulata* infection^11,43^. In view of the data obtained, we could conclude that the use of the live attenuated Egyptian cell line infected with *T. annulata* has potential efficacy in protecting both exotic and crossbred cattle against the clinical form of *T. annulata* even in highly endemic areas and should be recommended as a vaccine against tropical theileriosis in Egypt as in many other countries to overcome this problem in our Egyptian field. This is also consistent with previous studies that have recommended *T. annulata* attenuated cell line as a vaccine to control tropical theileriosis in many countries^11,22–24^. However, before mass production and field application, we recommend further studies to assess its polymorphism and interaction with wild circulating strains to ensure that vaccinated cattle are effectively immune to challenge with the same parasite strain and protected against a heterologous strain of this parasite as well as co-infections.

In this context, T-cells play a crucial role in both induction and maintenance of the immunity. It has been shown that the generation of cytotoxic T lymphocytes (CTL) is closely related to the control of the infection—macroschizont-infected cells are killed in an MHC class I restricted manner^42,44^. Any strain-specificity induced by immunization is likely to be manifested by CTL^45^. Besides CTLs, CD4 + T-cells also play an important role in protective immunity to *T. annulata* infection. They produce macrophage-activating cytokines such as IFN-gamma which produce mediators such as NO to destroy the intracellular schizonts. Based on the above, the protection observed in the animals after immunization with attenuated schizont in our study could also be mediated by CTL and CD4 + T cells and it is one of the recommended research points in our future studies to identify this role in detail. Moreover, this recommendation is supported by a recent study indicating that there is a great heterogeneity of the field *T. annulata* population in the same geographical area of our study^46,47^. These studies also recommended the production of cocktail vaccines including more than one isolate may be required as a further step to overcome the parasite’s heterogeneity in the future. Work is still ongoing to estimate the duration of immunization and the need for booster doses.

## Conclusion

In conclusion, Egyptian *T. annulata* attenuated cell line is efficient and can protect both exotic and cross breed cattle reared in endemic areas against Tropical theileriosis. By using this attenuated cell line as a vaccine, the case fatality rates will decrease dramatically. Although, the obtained results are promising, we still recommend more trials targeting different animals in different areas like Nile Delta and Lower Egypt because these areas may have different epidemiological status which may be affect the target animals, target age or time of vaccination.

## Patent

Patent number EG 2019121920A1 ‘Attenuated Tissue Culture Vaccine Against *Theileria annulate*—Egyptian strain submitted to Patent office, Academy of Scientific research and technology, Ministry of scientific research, Egypt*.*

## Methods

### Isolation of T. annulata infected cells

Heparinized blood was collected from a three-year-old cow naturally infected with *T. annulata.* This cow was admitted to the Teaching Veterinary Hospital, Faculty of Veterinary Medicine, Assiut University from EL-Ghanim district, Assiut Governorate. The animal exhibited typical clinical signs of tropical theileriosis. Its body temperature was 41.5 °C, with congested conjunctival mucosa and enlarged superficial lymph nodes. An aspirate was taken from the prescapular lymph nodes. Following sampling and diagnosis, the cow was treated with buparvaquone (MSD Animal health, New Jersey, USA) at 2.5 mg/kg body weight by deep intramuscular injection, followed by a second dose after 72 h. The animal was also treated with non-steroidal anti-inflammatory drugs (Butafenil, Vetoquinol, France) and antibiotics (Marbocyl 10%, Vetoquinol, France) to control possible secondary bacterial infections.

### Preparation and attenuation of T. annulata infected cell line

*T. annulata* strain Assiut is a single isolation initiated in 2015 from peripheral blood mononuclear cells (PBMC) of an ~ 3-year-old infected cow from El-Ghanayem, Assiut, Egypt, admitted to the Veterinary Teaching Hospital, Faculty of Veterinary Medicine, Assiut University and confirmed positive for* T. annulata* infection. Preparation of the PBMC from heparinized blood was performed by gradually transferring three mL of blood to a Falcon tube containing an equal amount of Ficoll® 400 (Sigma Aldrich, Germany) followed by centrifugation at 1800 rpm for 40 min. The PBMC layer was subsequently collected and transferred to a new Falcon tube in which it was washed three times with RPMI 1640 (Cat. No, BE12-115F, Lonza, Switzerland) with a centrifugation step at 2000 rpm for 10 min between washes. The pellet was subsequently resuspended in 2 mL complete RPMI 1640 media with 10% inactivated fetal calf serum (LSP, UK), 1% Amphotericin B (Lonza, Switzerland), 1% Gentamicin (Lonza, Switzerland), 2% Streptomycin and Penicillin (Lonza, Switzerland). Lymph node aspirates were washed three times in RPMI 1640 and resuspended in 2 mL complete media. Both samples were then transferred to two separate filter-cap tissue culture flasks 25 T containing 5 mL complete RPMI 1640 and were incubated with 5% CO_2_ at 37 °C. The media was changed every 72 h. *T. annulata* schizont-infected leukocytes grew continuously and were passaged under sterile laboratory conditions up to 114 serial passages. The obtained cell line was tested to confirm that it was free from any bacterial, mycoplasma or fungal contamination by the regular inoculation of 100 µL from the cell line on enriched 4% sheep blood agar (BioLab ’BAN20500’) and EcoBio, Sabouraud-Dextrose-Agar 4% PH Euro-USP, (BioLab ’ESDA20500’) and incubate the plates at 37 °C for 48 h and 25 °C for two week, respectively before inspection^48^. The DNA extracts from the cultures were tested using 18S rRNA and RLB assays, followed by sequencing^3^. The viability of the cell line was evaluated using Trypan blue (Lonza, Switzerland) exclusion counting before storage of each passage in liquid nitrogen and prior to preparation of the vaccine doses Cell viability ranged between 97 and 99% before storage and vaccination^22–24^.

### Evaluation of the appropriate dose and passage for vaccination

Different groups of exotic male calves, aged between 6 and 9 months, were used to evaluate the appropriate dose and passage for the vaccination trial. Each group consisted of three animals free of *T. annulata* infection, its antibodies, and ticks. These animals were kept in tick-free pens for one month as a pre-inoculation period, then inoculated with the attenuated *T. annulata* infected cell line and kept for a further three to four weeks as a post-inoculation period. Each group was injected subcutaneously in the middle third of the neck with a dose of 4 mL containing 500,000, or 1 × 10^6^ cells/ml, using different passages, including passages 45, 65, and 85, and observed for the development of clinical signs, including fever, respiratory, and/or ocular signs. Blood was routinely examined for infection 48–72 h after injection. Three blood samples were collected from each animal: one was collected directly from the ear vein for the preparation of Giemsa-stained thin blood smears; the second and third ones were collected from the jugular vein. The second one was EDTA blood collected for DNA extraction, followed by Tams-1 and 18S rRNA PCR, and the third blood sample was collected at 21 days post-inoculation for serum preparation and tested for the detection of the positive antibody against *T. annulata*^49^. These animals were then transported and kept in the field for natural challenges. From the data collected during these trials, we concluded that passage 85 at a dose of 4 ml (1 × 10^6^ cells/ml) was the highly immunogenic dose that could induce the strongest immune response without any adverse effects. It was able to protect the vaccinated animals during the natural challenge under field conditions, as we didn't observe any clinical signs other than a transient fever up to the time of slaughter (Table 4).

**Table 4:** Assessment of the appropriate dose and passage for vaccination.

No. of group	No. of animals	No. the used passage	Dose (The numbers of cells in 4 ml)	Clinical signs			Blood smears	Tams-1 PCR & 18SsrRNA	ELISA Cutoff*	ELISA Results
				Fever ≥ 40	Enlargement of the superficial lymph nodes	Respiratory/ Ocular lesions				
1	3	45	500.000 cells/ml	Positive	Enlarged	Positive	Positive	Positive	45.67%	Positive
2	3	45	1 × 10^6^ cells/ml	Positive	Enlarged	Positive	Positive	Positive		Positive
3	3	65	500.000 cells/ml	Positive	Enlarged	Moderate	Positive	Positive		Positive
4	3	65	1 × 10^6^ cells/ml	Positive	Enlarged	Positive	Positive	Positive		Positive
5	3	85	500.000 cells/ml	Negative	Negative	Negative	Negative	Positive		Positive
6	3	85	1 × 10^6^ cells/ml	Negative	Negative	Negative	Negative	Positive		Positive titer ≥ 100% PP

*The cutoff value set at 45.67% percentage positivity at sensitivity 92% and specificity 96% (Borstel -Research institute, Germany 2012).

### Experimental animals

Twenty-four exotic breed (Friesian) six-month old male calves were purchased from Assiut’s official governmental farm, which is located approx. 50 km north of Assiut city. Twenty-four crossbred male calves of the same age were purchased from a private farm in Arab EL-Awamer village, which is located approx. 75 km north to Assiut city.

### Preparation of animals for vaccination trail

All calves were kept in tick-free pens for 6–8 weeks prior to immunization. During this time, they were regularly observed and examined to confirm that they were free from any disease or parasitic infection. Blood samples from each animal were regularly collected and examined before and during the field challenge. Complete blood counts (CBC) and Giemsa-stained blood smears were made to assess the animal’s health status. PCR assays targeting the merozoite-piroplasm surface antigen 1 (Tams-1) of *T. annulata* and the 18S rRNA gene of *Babesia and Theileria* species were performed to test the animals for the presence of Babesia and Theileria infections. In addition to this, all animals were screened by an indirect TaSp-ELISA to check for previous *T. annulata* exposure prior to the start of the vaccination trial^8,50^.

### Immunization

Both the crossbred and exotic groups were randomly divided into two equal subgroups (vaccination and control groups). Each animal in the vaccination group was inoculated subcutaneously in the middle third of the neck with 4 mL (1 × 10^6^ cells/ml) of the passage 85 attenuated cell line. Animals in the control group were injected with RPMI 1640 medium only. Both groups were kept in tick-free enclosures for 3–4 weeks under continuous clinical, hematological, and parasitological monitoring, including molecular and serological testing as described above. Both groups were then transported to EL-Wady EL-Geded governorate, which is in the western desert plateau and occupies approximately 44% of the total area of the Arab Republic of Egypt^51^ and is considered an endemic area for *T. annulata*, with a prevalence rate of 63.6% based on molecularly confirmed infection^1^, and has two main tick species, *Hyalomma* spp. (*Hyalomma anatolicum and Hyalomma excavatum)*, the main vector of *T. annulata*^1,3,11^. All animals were kept under natural conditions at this site for three months to be subjected to a field challenge.

### Field monitoring

Calves were observed daily to assess the tick infestation rate and to monitor the development of clinical signs. Rectal temperature was recorded three times a day: before sunrise, at noon and after sunset. The mean of the three values was considered as the daily body temperature. Blood samples were collected from the jugular vein three times a week on ethylenediaminetetraacetic acid (EDTA) and examined for the presence of *T. annulata* developmental stages. Lymph node aspirates from animals that showed enlarged lymph nodes were collected and used for the preparation of Giemsa-stained lymph smears, which were subsequently examined microscopically. The parasitemia’s percentage in each animal was calculated using the following equation (Parasitemia % = (Number of the parasitized Red Blood corpuscles (RBCs)/12,500 RBCs × 100 which represent the total number of RBCs in 25:50 Microscopic Fields). Serum samples were collected every month for screening using the TaSp ELISA^9,25^. PCR was used for detection of *T. annulata* infection in the blood samples collected from animals before and after immunization and challenge in both groups^52,53^. Positive PCR products were purified using QIAGEN PCR purification Kit (Cat. No. 28104, Qiagen, UK) according to manufacturer’s instructions and sequenced in both directions using an ABI 310 Sequencer at the Molecular Biology Research & Studies Institute of Assiut University^54^. Sequences were subjected to BLAST similarity searches. A RLB assay for the simultaneous detection of Theileria/Babesia and Anaplasma/Ehrlichia was employed to determine if tick-borne pathogens other than *T. annulata* were present in the blood samples^2,3^.

### Necropsy finding

Post-mortem examinations were performed at the Department of Pathology and Clinical Pathology, Faculty of Veterinary Medicine, Assiut University, on calves that died during the experiment. Lymph nodes, liver, spleen lung and hearts were collected from the dead animals for further investigation^14^.

### Tick collection and identification

Ticks were manually collected from animals using a forceps and identified morphologically according to standard morphological keys^55^.

### Statistical analysis

General linear model analysis was conducted in R. Pairwise analyses were attached by least square means analyses for multiple comparisons under the lsmean package with Tukey adjustment. Significance levels were interpreted as: P-Value ≤ 0.00*** (Highly significant),0.001** (Moderate significant),0.01* (Mild significant), 0.05 (Non-significant)^56–58^.

### Ethical approval

The study is reported in accordance with ARRIVE guidelines. the working protocol, procedures, and all the experiments were ethically reviewed and approved by the Scientific Research Committee and Ethics Board of Assiut University, Egypt, approval number is IRB no: 04-2022-300018. The working protocol, procedures, and all the experiments conformed to recognized standards of Animal research applied by Assiut University and the animal welfare code in Egypt. Additional ethical approval was obtained from both Assiut governorate (Assiut University), 71526, (616, 24.02.2019) Egypt and New Valley Governorate (686, 2.3.2019) and the Veterinary authorities in the New Valley Governorate (485, 3.3.2019).

## Data Availability

All data generated or analyzed during this study are included in this submitted article.
